# Resilience, stress and anxiety in pregnancy before and throughout the pandemic: a structural equation modelling approach

**DOI:** 10.1007/s12144-022-03305-6

**Published:** 2022-06-09

**Authors:** Jose A. Puertas-Gonzalez, Carolina Mariño-Narvaez, Borja Romero-Gonzalez, Raquel Vilar-López, Maria Isabel Peralta-Ramirez

**Affiliations:** 1 Mind, Brain and Behaviour Research Center (CIMCYC), Granada, Spain; 2 Personality, Assessment and Psychological Treatment Department, Faculty of Psychology, University of Granada, Granada, Spain; 3 Psychology Department, Faculty of Education, University of Valladolid, Campus Duques de Soria, Soria, Spain

**Keywords:** SEM, Pandemic, COVID-19, Pregnancy, Resilience, Stress, Anxiety

## Abstract

The present study explored and compared the link between resilience and pregnancy-related stress, perceived stress, and anxiety, employing two structural equation models. One model focused on pregnant women before the outbreak of the pandemic, and the other on pregnancies throughout the pandemic. For this purpose, a total sample of 690 women during their pregnancy were collected: the Pre-Pandemic Group (P-PG) was composed of 341 pregnant women evaluated prior to the pandemic; and 349 pregnant women assessed at the time of the pandemic constituted the Pandemic Group (PG). The resilience, pregnancy-related stress, perceived stress, and anxiety symptomatology of the women were assessed. For both samples, resilience was found to lower levels of pregnancy-specific stress, as well as general perceived stress, and anxiety symptomatology. Furthermore, pregnancy-specific stress and perceived stress showed a covariance relationship and, that these, in turn, increased the anxiety. Moreover, the PG showed greater levels of pregnancy-specific stress, anxiety, somatisations, and obsessions-compulsions, while the P-PG presented higher perceived stress levels.

## Introduction

Due to COVID-19, a worldwide pandemic was announced to start in the month of March 2020 (WHO, 2020). Because of the health threat, economic ramifications, and disruption of everyday routines, the COVID-19 has had an enormous impact on individuals and can be considered a worldwide stressor. In addition to the death toll, the pandemic has caused widespread agitation and concern among the general population, due to fears of contagion and its consequences, bringing about a rise in various psychopathological symptoms such as anxiety (Wang et al., 2020).

A particularly vulnerable population group is pregnant women. Indeed, their levels of pregnancy-specific stress, as well as general stress and anxiety were found to have increased (Boekhorst et al., 2021; Lebel et al., 2020; Medina-Jimenez et al., 2020; Romero-Gonzalez et al., 2021). Pregnancy-related stress along with general stress has a strong comorbidity during pregnancy (Alderdice et al., 2012; Romero-Gonzalez et al., 2020a), and were also shown to be predictors of psychopathological symptomatology, including anxiety, at various times during pregnancy, before and at the time of the pandemic (Moyer et al., 2020; Peñacoba-Puente et al., 2016; Romero-Gonzalez et al., 2020a). This symptomatology, when suffered persistently throughout pregnancy, raises the probability of developing postpartum depression, as well as the risk of preeclampsia and hypertension, miscarriages, the need for instrumented deliveries, preterm births, low birth weight and low scores on the Apgar test (Accortt et al., 2015; Bayrampour et al., 2016; Caparros-Gonzalez et al., 2017; Coussons-Read, 2013; Qu et al., 2017; Rondó et al., 2003; Romero-Gonzalez et al., 2019; Stein et al., 2014). Moreover, those symptoms could impact on the mother's state of mind and on the development of the foetus because they can lead to alterations regarding physical activity, nutrition and sleep (Coussons-Read, 2013). Furthermore, offspring of women that experience large amounts of stress at the time of their pregnancy have a greater likelihood of developing both cognitive and behavioural deficits, and an increased likelihood of suffering from mental health problems further on in life (Glover, 2014; MacKinnon et al., 2018; Van den Bergh et al., 2018, 2020).

Given such negative consequences, resilience acts as an important buffer against psychological distress both in the population in general (Oken et al., 2015), and in women during their pregnancy (García-León et al., 2019). Resilience indeed represents an individual’s set of personal resources allowing them to optimally face stressors and difficulties (Fletcher & Sarkar, 2013; Newman, 2005). Thus, several investigations have discovered a negative association between resilience and anxiety during pregnancy (Lubián López et al., 2021), and between resilience towards stress and anxiety in other populations at the time of the pandemic (Braun-Lewensohn et al., 2021; Satici et al., 2020: Wang et al., 2021). Additionally, previous researches carried out before the pandemic have proven the protective role of resilience regarding these variables in the perinatal stage (Armans et al., 2020; García-León et al., 2019).

Nevertheless, to date no study has analysed and compared resilience's position as a buffer for psychological stress and anxiety in pregnant women at two different moments in time: before society faced a devastating event such as the COVID-19 pandemic, and when it erupted. Hence, the purpose of this investigation was to explore and compare the link of resilience to general perceived stress, pregnancy-specific stress and anxiety, and to study the relationships of these variables, using two structural equation models. The first model focused on pregnant women before the pandemic, and the second on women that were pregnant at the time of the COVID-19.

The first hypothesis of the structural equation models is that resilience negatively influences pregnancy-related stress, and also perceived stress and anxiety symptomatology.

A second hypothesis is that pregnancy-related stress and perceived stress present a correlation and these, in turn, positively influence anxiety symptomatology.

Finally, if the hypothesised models present a good fit, the variables included in the samples will be compared to check whether there are differences between the two groups.

## Methods

### Participants

The sample was composed of 690 pregnant women, which were split into two different subgroups: Pre-Pandemic Group (P-PG), formed by 341 (49.4%) pregnant women evaluated before the COVID-19 pandemic (mean age = 33.35, SD = 4.53), and Pandemic Group (PG), made up of 349 (49.6%) pregnant women assessed throughout the pandemic (mean age = 33.9, SD = 4.15).

All participants included in the research were briefed on the procedure and objectives and participated on a voluntary basis. The following were the conditions for participating in the study: knowing how to write and read properly in Spanish; being at least 18 years old; and being pregnant. On the other hand, the exclusion criterion was active treatment with psychopharmaceuticals.

The present research was approved by the ethics committee of the University of Granada (reference 881; and reference 1518/CEIH/2020).

### Instruments

Obstetric and socio-demographic variables were gathered and, in parallel, the subsequent psychological assessment tools were applied:

- The Connor-Davidson Resilience Scale (CD-RISC) (Connor & Davidson, 2003) in the Spanish abbreviated form (Notario-Pacheco et al., 2011): it was employed to estimate the level of resilience. It measures the ability to deal with different life circumstances such as diseases, changes, stress, failures, personal difficulties and feelings of grief. It is responded on a Likert scale with 5 alternatives from 0 = "almost never" to 4 = "almost always", and is composed as a set of 10 items. The Cronbach’s alpha was 0.88 in this research.
- The Perceived Stress Scale (PSS) (Cohen et al., 1983; Spanish validation by Remor, 2006): this instrument reports on perceived general stress in the past month. PSS provides scores between 0 and 56 (greater ratings indicate greater perceived stress) and it is made up of 14 items scored on a 5-point Likert scale (“very often”, “often”, “once in a while”, “almost never”, “never”). Its Cronbach’s alpha was 0.73 in this research.
- The Prenatal Distress Questionnaire (PDQ) (Yali & Lobel, 1999; Spanish validation by Caparros-Gonzalez et al., 2019): the PDQ consists of a scale of 12 items for evaluating pregnancy-related stress (g., worries regarding health problems, childbirth, body symptoms, corporal alterations and/or the baby's general health). The answers of this instrument are provided through a Likert-type scale from 0 = “not at all” to 4 = ” very much”. Its Cronbach’s alpha was 0.77 in this study.
- The Symptom Checklist-90-Revised (SCL-90-R) (Derogatis, 1994; Spanish validation by Caparrós-Caparrós et al., 2007): were applied to measure the level of anxiety symptoms. Specifically, the scales in this instrument assessing anxiety disorders are the obsessions and compulsions dimension, the anxiety dimension and the phobic anxiety dimension. These use a Likert scale with 5 answer alternatives from 0 = “never” to 4 = ” extremely”. In addition, we added the somatisation scale because of their link with anxiety and the other measures included in the models. Thus, some studies have shown that COVID-19 has enhanced somatisations along anxiety in the population as a whole (Wang et al., 2020), while other pre-pandemic research focusing on pregnant women found correlations between resilience and stress with somatisations and anxiety (García-León et al., 2019; Scharlau et al., 2018). The 4 dimensions had an acceptable reliability, the Cronbach’s alpha ranging from 0.75 to 0.84 for all four dimensions in this study.

### Procedure

The two groups of participants in this research were enlisted at the San Cecilio University Hospital and at the Góngora and Mirasierra health centres in Granada, Spain. When potential participants went to their appointment with the midwife for their pregnancy follow-up, they were provided with study information and were offered the possibility to participate in the research. Subsequently, the contact information of the women that agreed to their participation in the research were collected and the survey questionnaires were submitted to them online. The questionnaires were all done through Google Forms. At that time, they were also asked to inform us of any potential persons interested in participating to include them in the study.

The P-PG participants were recruited and evaluated between late 2017 and early 2020, as they formed part of an earlier research study entitled Gestastress. In addition to the recruitment through their medical practitioners, PG members were also captured through several social media networks of pregnant women (via internet forums, WhatsApp and Facebook) and assessed between March 2020 and March 2021 at the time of the pandemic. Other studies have used two groups from different years to evaluate the worldwide pandemic disease's impact on prenatal mental health (Puertas-Gonzalez, et al., 2021; Zanardo et al., 2020).

### Data analysis

First, the two groups were compared to examine if they were evenly homogeneous in relation to primary sociodemographic and obstetrical characteristics. For continuous variables, t-test was applied while the Chi-square test was performed in order to analyse qualitative variables.

Subsequently, with the aim of checking whether the latent variable presented an adequate goodness-of-fit for constituent factors (anxiety, phobic anxiety, somatisation and obsessive–compulsive) in both groups, preliminary confirmatory factor analyses (CFA) were carried out. The factor load for each factor was set to at least 0.50, in order to ensure a good fit (Hair et al., 1998). Additionally, before carrying out the Structural Equation Modelling (SEM), zero-order correlations between all variables in the models were also calculated.

Then, the SEM was performed with the Maximum Likelihood Estimator (ML), considering the appropriate statistical requirements to be met to guarantee a good model fit. Thus, for both models, cut-off points for the comparative fit index (CFI) and the Tucker-Lewis index (TLI) were set at > 0.95. While for the standardised root mean square residual (SRMR) was set at < 0.08 and for the root mean square error of approximation (RMSEA) was also set at < 0.08 (Hu & Bentler, 1999).

Finally, both groups were compared in relation to the psychological variables measured. In addition, for continuous variables in which statically significant discrepancies were identified, the effect size was calculated on the basis of Cohen’s d, and then interpreted according to values proposed by Cohen (1988): large effect size (≥ 0.80); median effect size (≥ 0.50); and small effect size (≥ 0.20).

For the CFA and SEM analysis, the software R 4.0.1 (R Core Team, 2020) was used, implementing the “lavaan” package (Rosseel, 2012).

## Results

### Sample description

Of the 341 participants who formed the P-PG, 20 (5.9%) were in their first trimester of pregnancy (weeks 1–12), 175 (51.3%) in their second trimester (weeks 13–26), and 146 (42.8%) in their third trimester (weeks 27–40). In turn, of the 349 participants in the PG group, 32 (9.3%) were in their first trimester of pregnancy, 167 (47.9%) in their second trimester, and 150 (43%) in their third trimester. No significant differences were found regarding the P-PG and PG for the primary socio-demographic and obstetric variables. These results are set out in Table 1.

**Table 1:** Analysis of obstetric and socio-demographic variables

		P-PG (n = 341) M(SD)	PG (n = 349) M(SD)	*t*	*p*
Age of participants		33.35 (4.53)	33.96 (4.15)	1.836	.067
		P-PG (n = 341) n(%)	PG (n = 349) n(%)	χ2	*p*
Socio-demographic characteristic					
Current partner	No	8 (2.3%)	14 (4%)	1.550	.213
	Yes	333 (97.7%)	335 (96%)		
Nationality	Spanish	293 (85.9%)	305 (87.4%)	1.364	.505
	Inmigrant	48 (14.1%)	44 (12.6%)		
Education level	Primary school	5 (1.5%)	2 (0.6%)	3.892	.143
	Secondary school	95 (27.9%)	80 (22.9%)		
	University	240 (70.6%)	267 (76.5%)		
Obstetric information					
Trimester of pregnancy	1º	20 (5.9%)	32 (9.2%)	2.918	.232
	2º	175 (51.3%)	167 (47.9%)		
	3º	146 (42.8%)	150 (43%)		
Pregnancy method	Spontaneous	298 (87.4%)	309 (88.5%)	.215	.643
	Fertility treatment	43 (12.6%)	40 (11.5%)		
Previous miscarriages	0	202 (59.6%)	228 (65.3%)	8.921	.063
	1	76 (22.3%)	84 (24.1%)		
	2	40 (11.7%)	25 (7.2%)		
	3	13 (3.8%)	6 (1.7%)		
	≥ 4	10 (2.9%)	6 (1.7%)		
Previous children	0	192 (56.3%)	207 (59.3%)	5.521	.063
	1	118 (34.6)%	126 (36.1%)		
	≥ 2	31 (9.1%)	16 (4.6%)		
Primiparous	No	179 (52.5%)	160 (45.8%)	3.049	.081
	Yes	162 (47.5%)	189 (54.2%)		
Risk pregnancy	No	274 (80.4%)	274 (84.2%)	1.792	.181
	Yes	67 (19.6%)	55 (15.8%)		

*P-PG* Pre-Pandemic Group, *PG* Pandemic Group

### Confirmatory factor analysis results

**Fig. 1:**
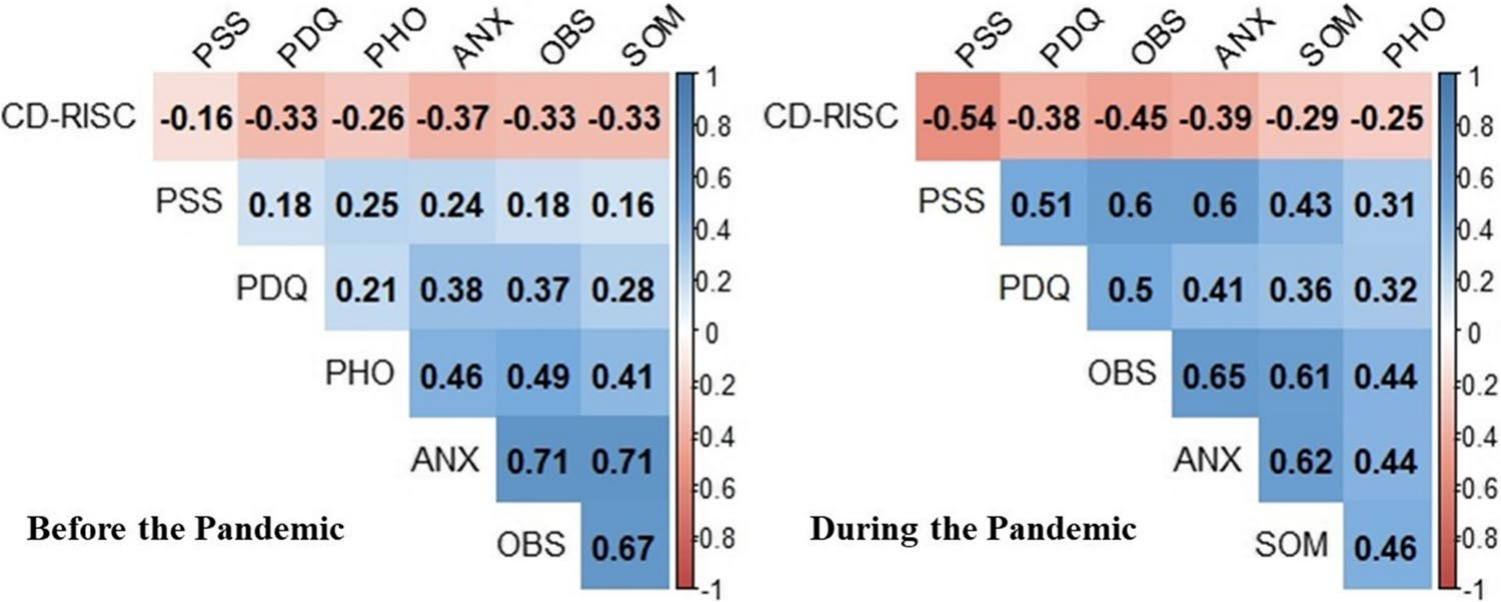
Zero-ordered correlations for all observable variables in the models of both groups. Note: Significant correlations at *p* < .05 are highlighted in red (negative) or blue (positive); CD-RISC = Connor Davidson Resilience Scale; PSS = Perceived Stress Scale; PDQ = Pregnancy Distress Questionnaire.; ANX = SCL-90-R Anxiety Dimension; OBS = SCL-90-R Obsession-Compulsion Dimension; SOM = SCL-90-R Somatization Dimension; PHO = SCL-90-R Phobic Anxiety Dimension

For each group, a CFA analysis was conducted to check whether the latent variable of anxious symptomatology showed adequate goodness of adjustment in terms of the all factors that comprise it (anxiety, phobic anxiety, somatisation and obsessive–compulsive). In relation to P-PG, the CFA showed an acceptable fit for anxiety symptomatology: χ2 = 6.032 with 2 degrees of freedom (*p* = 0.049); CFI = 0.99; TLI = 0.96; RMSEA = 0.07 (90% CI: 0.04, 0.15; *p* = 0.195); SRMR = 0.02. With regard to PG a good fit was also obtained: χ2 = 1.215 with 2 degrees of freedom (*p* = 0.545); CFI = 1.00; TLI = 1.01; RMSEA = 0.01 (90% CI: 0.01, 0.09; *p* = 0.763); SRMR = 0.01. In both groups with standardized factor loadings for the four variables > 0.50. Therefore, the latent variable of anxiety symptomatology met the criteria for inclusion in the models for both groups. Moreover, all observable variables were also subjected to zero-order correlations (Fig. 1).

### Structural equation modelling results

**Fig. 2:**
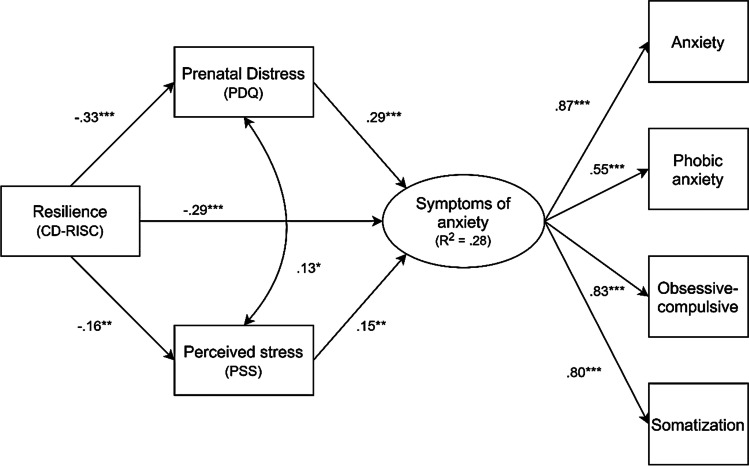
Structural equation model with standardized path coefficients predicting anxiety symptomatology in the Pre-Pandemic Group. The statistical values of the latent variable anxiety symptomatology correspond to the standardized factor loadings of the indicators. *Note:* *** p < .001; ** p < .01; * p < .05

In relation to the P-PG, the SEM model proposed presented a good adjustment: χ2 = 20.020 with 11 degrees of freedom (*p* = 0.045); CFI = 0.99; TLI = 0.98; RMSEA = 0.05 (90% CI: 0.01, 0.08); SRMR = 0.03. Resilience variable was shown to be negatively associated with anxiety symptoms (*β* = -0.29; SE = 0.07; *p* = 0.001). Furthermore, anxiety symptoms were positively influenced by perceived stress (β = 0.15; SE = 0.06; *p* = 0.003) and pregnancy-specific stress (*β* = 0.29; SE = 0.07; *p* = 0.001). In turn, resilience negatively influenced perceived stress (*β* = -0.16; SE = 0.06; *p* = 0.004) and pregnancy-specific stress in the SEM (*β* = -0.33; SE = 0.05; *p* = 0.001). Moreover, the perceived stress and the pregnancy-specific stress presented a significant correlation (φ = 0.13; SE = 0.05; *p* = 0.017). Finally, resilience indirectly negatively influenced anxiety symptomatology through perceived stress (*β* = -0.05; SE = 0.02; *p* = 0.012), as well as through pregnancy-specific stress (*β* = -0.10; SE = 0.03; *p* = 0.001). Thus, this model explained 28% of the variance, through R^2^, of anxious symptomatology in pregnancy prior the COVID-19 (Table 2 and Fig. 2).

**Table 2:** Results of structural equation modelling

Model	*β/* φ	SE	*p*	χ2	df	CFI	TLI	RMSEA	SRMR	R^2^
Model 1: Pre-Pandemic Group				20.020	11	0.99	0.98	0.05	0.03	0.28
CD-RISC → PDQ	-0.33	0.05	.001**							
CD-RISC → PSS	-0.16	0.06	.004**							
CD-RISC → Anxiety	-0.29	0.07	.001**							
PDQ ↔ PSS	0.13	0.05	.017*							
PDQ → Anxiety	0.29	0.07	.001**							
PSS → Anxiety	0.15	0.06	.003**							
CD-RISC → PDQ → Anxiety	-0.10	0.03	.001**							
CD-RISC → PSS → Anxiety	-0.05	0.02	.012*							
Model 2: Pandemic Group				31.574	11	0.98	0.96	0.07	0.03	0.54
CD-RISC → PDQ	-0.38	0.05	.001**							
CD-RISC → PSS	-0.55	0.04	.001**							
CD-RISC → Anxiety	-0.18	0.08	.023*							
PDQ ↔ PSS	0.38	0.04	.001**							
PDQ → Anxiety	0.24	0.08	.001**							
PSS → Anxiety	0.51	0.09	.001**							
CD-RISC → PDQ → Anxiety	-0.05	0.03	.030*							
CD-RISC → PSS → Anxiety	-0.06	0.04	.026*							

*SE* standard error, *CD-RISC* The Connor-Davidson Resilience Scale, *PDQ* The Pregnancy Distress Questionnaire, *PSS* The Perceived Stress Scale

* =  ≤ .05; ** = p ≤ .01

**Fig. 3:**
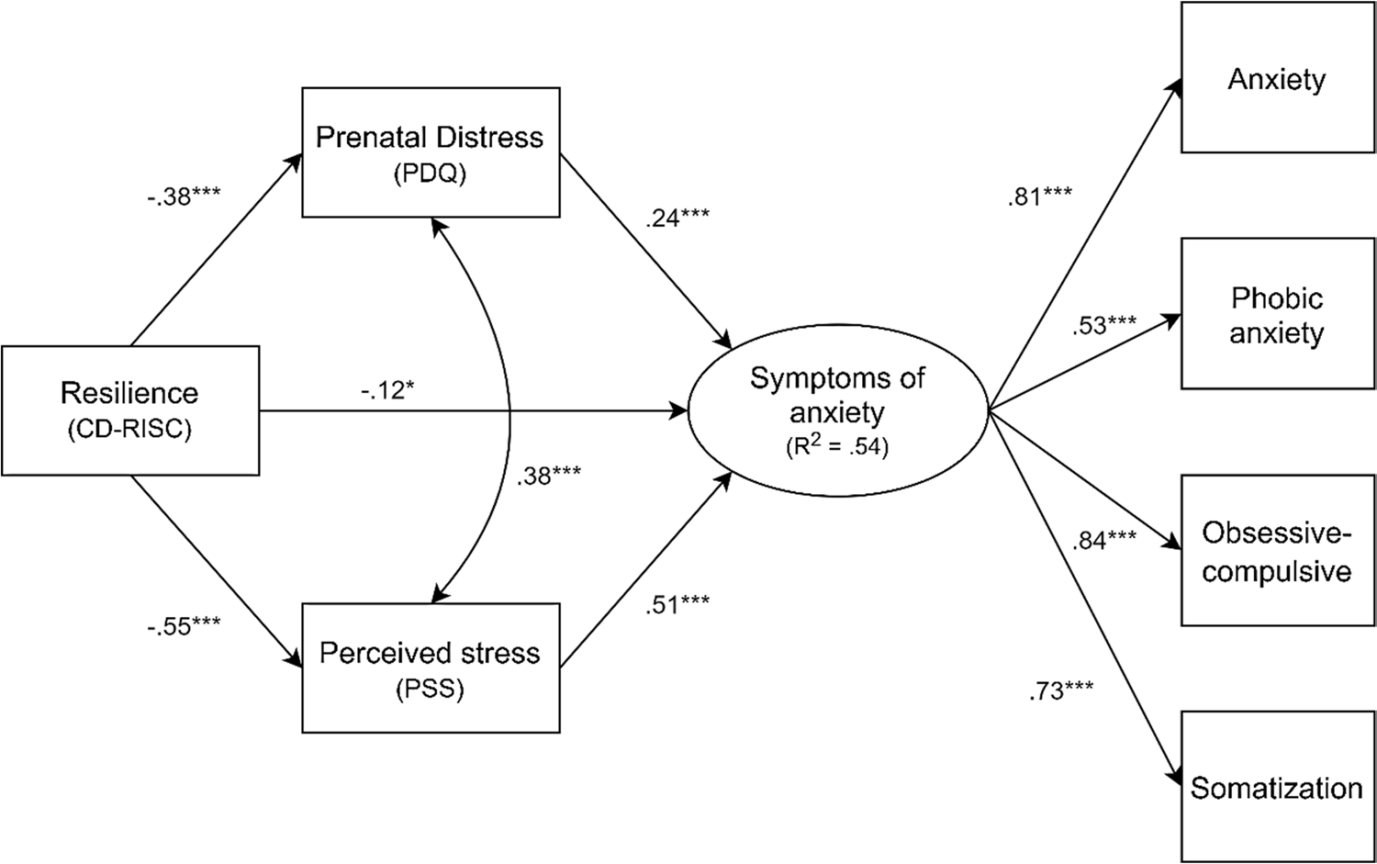
Structural equation model with standardized path coefficients predicting anxiety symptomatology in the Pandemic Group. The statistical values of the latent variable anxiety symptomatology correspond to the standardized factor loadings of the indicators. *Note:* *** p < .001; ** p < .01; * p < .05

With respect to the PG, the SEM model also presented a good fit: χ2 = 31.574 with 11 degrees of freedom (*p* = 0.001); CFI = 0.98; TLI = 0.96; RMSEA = 0.07 (90% CI: 0.05, 0.10; *p* = 0.093); SRMR = 0.03. Anxiety symptoms were negatively influenced by resilience (*β* = -0.18; SE = 0.08; *p* = 0.023) and positively influenced by perceived stress (*β* = 0.51; SE = 0.09; *p* = 0.001) and pregnancy-specific stress (*β* = 0.24; SE = 0.08; *p* = 0.001). Moreover, resilience presented a negative influence on the perceived stress variable (*β* = -0.55; SE = 0.04; *p* = 0.001) and pregnancy-specific stress (*β* = -0.38; SE = 0.05; *p* = 0.001). The latter in turn showed a significant correlation (φ = 0.38; SE = 0.04; *p* = 0.001). Finally, resilience negatively influenced anxiety symptomatology indirectly for perceived stress (*β* = -0.06; SE = 0.04; *p* = 0.026) and pregnancy-specific stress (*β* = -0.05; SE = 0.03; *p* = 0.030). Overall, this model explained 54% of the variance, through R^2^, of the anxious symptoms in pregnancy at the time of the pandemic (Table 2 and Fig. 3).

### Differences in resilience, anxiety symptomatology and stress between groups

The comparative analysis between groups using *Student's t* showed statistically significant differences regarding the SCL-90-R dimensions: obsession-compulsion [*t* (688) = 2,589; *p* = 0.010; *d* = 0.20], anxiety [*t* (679) = 3.059; *p* = 0.002; *d* = 0.23] and somatisations [*t* (688) = 2.676; *p* = 0.008; *d* = 0.20]. In addition, statistically significant differences were found for pregnancy-specific stress [t (681) = 5,323; *p* = 0.001; *d* = 0.41] and perceived stress [*t* (504) = -4.808; *p* = 0.001; *d* = 0.36]. PG scored higher in all the above variables except for perceived stress, where it scored lower than the P-PG. However, there were no significant differences regarding P-PG and PG on either the resilience variable or the phobic anxiety. These results are set out in Table 3.

**Table 3:** Comparison of means of psychological variables by Student's t-test

	P-PG (n = 341) M(SD)	PG (n = 349) M(SD)	*t*	*p*	Cohen´s d
CD-RISC	28.14 (5.87)	27.58 (6.55)	-1.194	.233	0.09
PSS	26.45 (4.40)	23.83 (9.16)	-4.808	.001**	0.36
PDQ	14.54 (6.18)	17.22 (6.99)	5.323	.001**	0.41
SCL-90-R Anxiety	62.79 (29.28)	69.32 (26.71)	3.059	.002**	0.23
SCL-90-R Phobic anxiety	57.10 (35.99)	60.51 (34.74)	1.267	.206	0.10
SCL-90-R Obsession-compulsion	68.06 (26.90)	73.23 (25.55)	2.589	.010**	0.20
SCL-90–R Somatisation	61.44 (25.05)	66.52 (24.88)	2.676	.008*	0.20

*P-PG* Pre-Pandemic Group, *PG* Pandemic Group, *CD-RISC* The Connor-Davidson Resilience Scale, *PDQ* The Pregnancy Distress Questionnaire, *PSS *The Perceived Stress Scale

* =  ≤ .05; ** = p ≤ .01

## Discussion

The objective of this study was to examine and compare the association between pregnancy-related stress, anxiety and resilience in pregnant women. For this aim, two structural equation models were carried out, one with participants prior to the pandemic and the other with women whose pregnancies occurred during the pandemic. Two hypotheses were raised. According to the first, resilience would negatively influence perceived stress and pregnancy-related stress as well as anxiety symptomatology in both groups. The second hypothesis was that, in turn, perceived stress and pregnancy-specific stress would show a positive relationship with anxiety symptomatology, and the latter would present a covariance relationship. This hypothesis was fulfilled, since all the relationships proposed at the beginning were found in both groups, based on the two structural equations models.

In relation to the information provided by the structural equations model of pregnant women during the pandemic, resilience was found to present a negative relationship with anxiety during pregnancy. These findings support those presented by Lubián López et al. (2021), in which they found the same negative relationship in pregnant women. In addition, it is worth mentioning that our results are in line with those of other authors who used structural equations models during the pandemic in other populations. Thus, they are consistent with the results of Wang et al., 2021, which found a direct negative relationship of resilience to stress and anxiety in a sample of medical personnel in a hospital in Wuhan, Hubei Province's provincial capital (China)—the location of the first outbreak of COVID-19. They also support the results of Rodríguez-Hidalgo et al. (2020), who showed a direct relationship between stress and anxious symptoms in university students throughout the COVID-19, again through a structural equation model. Therefore, the results demonstrate how resilience plays a protective role in pregnant women in times of crisis in the face of stress, pregnancy worries and anxiety symptomatology during the pandemic. This may be because resilience is defined as the psychological resources that allow a person to cope optimally with changes and adversities (Fletcher & Sarkar, 2013; Newman, 2005), and resilient people would present more adaptive coping. In turn, a deficit in personal resources for coping with adversity would lead to greater levels of psychological distress, leading to a sub-optimal adaptation to changes arising from the pandemic, such as lockdowns or mobility restrictions. These results imply that resilience can prevent the long-term negative effects of psychological stress and long-term anxiety, such as postpartum depression or the baby’s low birth weight (Caparros-Gonzalez et al., 2017; Coussons-Read, 2013; Rondó et al., 2003).

With respect to the results provided by the model of pregnant women before the pandemic, our findings also globally support previous studies showing how resilience can diminish stress as well as anxiety in the perinatal stage (García-León et al., 2019). Thus, our findings are in line with those provided by structural equation models in other studies. For example, they support those found by Armans et al. (2020), who showed that resilience negatively influenced pregnancy-specific stress, or those found by Peñacoba-Puente et al. (2016), which demonstrated how pregnancy worries had an impact on anxiety symptoms before the pandemic. The results provided by this model showed that resilience also acts as a buffering factor in relation to stress, pregnancy worries and anxious symptoms in women during their pregnancy before the COVID-19, and not only in times of crisis or great adversity. Therefore, resilience is also a protector against daily stress during pregnancy, e.g., attending follow-up medical appointments, psychosocial changes such as sick leave, delegating responsibilities regarding household tasks, etc. Nevertheless, this is the first study conducted with pregnant women before and at the time of the pandemic, and the first to investigate the connection between these factors using two structural equation models.

The results that showed an increased symptomatology of anxiety and pregnancy-related stress during the COVID-19 are in agreement with the studies that found such an increase in pregnancy throughout the pandemic (Boekhorst et al., 2021; Hessami et al., 2020; Lebel et al., 2020; Wu et al., 2020). The increase in anxiety, as well as the increase in prenatal worries, may be due to different factors arising from the pandemic, such as: fear of the disease's spread and possible negative effects on health and foetus; fear of losing loved ones; financial worries and the loss of direct social contacts due to the restrictions aimed at controlling contagion (Wang et al., 2020).

Nevertheless, in our study, we found that pregnant women before the pandemic reported greater levels of perceived stress compared to pregnant women during the pandemic. These results are contrary to those of Medina-Jimenez et al. (2020), who had previously found increased stress in women who were pregnant throughout the pandemic in Mexico. It is worth noting, however, that the present study has substantial differences with this latter one: the earlier investigation did not involve a control group before the pandemic and the present work was conducted in Spain, where the restrictions and lockdowns to stop the contagion were different. In addition, in the current investigation, the sample was recruited over a longer period during the pandemic. The causes for the lower levels of perceived stress in pregnancies throughout the pandemic may be multifactorial. One reason for this decrease in stress may be the increase in time stayed at home during periods of lockdown and the reduction of daily stressors such as: visits to the supermarket and other stores during the week; activities and/or presential courses; presential work; events and/or social commitments, etc. On the other hand, the promotion of teleworking and the flexible hours that often result from it may also have contributed to reducing the stress levels perceived by women pregnant during the pandemic.

In general terms, resilience, pregnancy-specific stress, and general stress better predicted the anxiety symptomatology appearing in the wake of the pandemic. These results may be due to differences with respect to these variables between groups, as pregnant women at the time of the pandemic showed greater levels of pregnancy-specific stress, anxiety, somatisation, obsessions-compulsions and similar levels of resilience. Before the pandemic, however, the pregnant women showed greater levels of perceived stress. These differences resulted not only in a stronger relationship in the pandemic group between resilience and perceived stress, but also between perceived stress and anxiety symptoms. This could indicate that in the pre-pandemic group, there could be other factors influencing perceived stress levels that would not be influencing the pandemic group, e.g. work stress, less time with a partner, less free time, etc. On the other hand, the raised levels of anxiety in the pandemic group could be a reflection of the increased pregnancy-specific stress found; as it has a stronger relationship with it compared to the pre-pandemic group, and could be due to pandemic-related concerns, such as fear of contagion and disruption of the gestational process. Thus, while exhibiting the same levels of resilience, variations in perceived stress and pregnancy-related stress show that they are influenced by different contextual factors in the two groups, reflecting different relationships with resilience itself, as well as with anxious symptoms.

A first conclusion is that resilience, stress and pregnancy worries better explained anxiety symptoms during the pandemic than before the pandemic. In addition, resilience played an important buffer role against general stress, pregnancy-related worries and anxiety symptomatology at both moments in time. Second, the pandemic may have increased pregnant women’s levels of anxiety and pregnancy-specific stress because of infection fear and the possible negative implications for them and their babies, in addition to uncertainties regarding the future. On the other hand, throughout the pandemic, women in the gestational period had lower levels of perceived stress, possibly due to reduced daily stress resulting from lockdowns and restrictions and increased hours at home. Based on all the above, this study has significant clinical implications: it is necessary to promote tools that have been shown to be effective at increasing resilience and reducing stress in pregnant women, thus preventing increases in anxiety symptoms in crisis situations (Puertas-Gonzalez et al., 2021; Romero-Gonzalez et al., 2020b).

### Strengths

A notable strength of this work was the inclusion of two samples from two different temporal and contextual moments (before and at the time of the pandemic).

### Limitations

Despite the findings, there are some limitations to this research. Firstly, as the instruments used for the assessment were sent online to the participants and therefore there was no control by a researcher at the time of completion, we cannot ensure that all questionnaires have been completed by pregnant women. However, as participants were not paid or rewarded for completing the questionnaire, and as it was a long questionnaire with a duration of 30–40 minutes, it was assumed that the people who completed the questionnaire were pregnant women. Secondly, no participant follow up was conducted to verify whether the results persisted over other periods, for example, during the postpartum period, so we propose this for future research.

Finally, given that we have demonstrated relationships between resilience and stress and anxiety in pregnancy, both in crisis situations and in normal life contexts, it would be highly interesting for future studies to test whether these relationships are the same for each trimester of pregnancy, as this would have implications for planning a specific psychological intervention for this population.

## Data Availability

The datasets generated during and/or analysed during the current study are available from the corresponding author on reasonable request.
